# Triple negative breast cancer: looking for the missing link between biology and treatments

**DOI:** 10.18632/oncotarget.5306

**Published:** 2015-08-30

**Authors:** Giuseppe Palma, Giuseppe Frasci, Andrea Chirico, Emanuela Esposito, Claudio Siani, Carmela Saturnino, Claudio Arra, Gennaro Ciliberto, Antonio Giordano, Massimiliano D’Aiuto

**Affiliations:** ^1^ S.S.D. “Sperimentazione Animale”, National Cancer Institute, IRCCS, “Fondazione Pascale”, Naples, Italy; ^2^ Division of Breast Surgery, Department of Breast Disease, National Cancer Institute, IRCCS, “Fondazione Pascale”, Naples, Italy; ^3^ Sbarro Institute for Cancer Research and Molecular Medicine, Center for Biotechnology, Temple University, Philadelphia, PA, USA; ^4^ Department of Pharmacy and Biomedical Science, University of Salerno, Fisciano, Italy; ^5^ National Cancer Institute, IRCCS, “Fondazione Pascale”, Naples, Italy; ^6^ Department of Psychology of Developmental and Socialisation Processes, “La Sapienza” University of Rome, Rome, Italy; ^7^ Department of Medicine, Surgery and Neuroscience, “University of Siena”, Siena, Italy

**Keywords:** breast cancer, triple negative, oncology, treatments, biology

## Abstract

The so called “Triple Negative Breast Cancer” (TNBC) represents approximately 15-20% of breast cancers. This acronym simply means that the tumour does not express oestrogen receptor (ER) and progesterone receptor (PR) and does not exhibit amplification of the human epidermal growth factor receptor 2 (HER2) gene. Despite this unambiguous definition, TNBCs are an heterogeneous group of tumours with just one common clinical feature: a distinctly aggressive nature with higher rates of relapse and shorter overall survival in the metastatic setting compared with other subtypes of breast cancer. Because of the absence of well-defined molecular targets, cytotoxic chemotherapy is currently the only treatment option for TNBC.

In the last decades, the use of more aggressive chemotherapy has produced a clear improvement of the prognosis in women with TNBC, but this approach results in an unacceptable deterioration in the quality of life, also if some support therapies try to relieve patients from distress. In addition, there is the general belief that it is impossible to further improve the prognosis of TNBC patients with chemotherapy alone. In view of that, there is a feverish search for new *“clever drugs”* able both to rescue chemo-resistant, and to reduce the burden of chemotherapy in chemo-responsive TNBC patients.

A major obstacle to identifying actionable targets in TNBC is the vast disease heterogeneity both inter-tumour and intra-tumour and years of study have failed to demonstrate a single unifying alteration that is targetable in TNBC. TNBC is considered the subtype that best benefits from the neoadjuvant model, since the strong correlation between pathological Complete Response and long-term Disease-Free-Survival in these patients.

In this review, we discuss the recent discoveries that have furthered our understanding of TNBC, with a focus on the subtyping of TNBC. We also explore the implications of these discoveries for future treatments and highlight the need for a completely different type of clinical trials.

## INTRODUCTION

Breast cancer (BC) is the leading cause of death from cancer among women and accounts for about 30% of all estimated new cancer cases in this population. In the last decade, the incidence rate was substantially stabilized in Western while it is dramatically growing in developing countries [1]. Worldwide the incidence of BC is rising up in the whole female population, but the growth rate is higher in young women in comparison to older ones [2]. Substantial progress has been made in the management of breast cancer, both from clinical and new supportive point of view [3] and this, together with the results obtained in the early diagnosis, has been reflected in substantial improvements in breast cancer mortality over the last decades [4]. Therapeutic improvements have come from the development of combination chemotherapy regimens, endocrine therapies, and HER2-targeting therapies. Additional steady improvements in outcome, both in the adjuvant and metastatic settings, can be anticipated for both oestrogen receptor ER-positive breast cancer and for HER2-positive cancers [5], given the progress currently being made with targeted therapies for these breast cancer subtypes. For patients with triple-negative breast cancers (TNBC), however, it is likely that the improvements in survival have currently plateaued. Although the outcome of patients with this disease has significantly improved with adjuvant chemotherapy, which reduces the risk of death by approximately 50%, there has been limited progress in incorporating additional systemic therapies into the management of TNBCs [6, 7]. Consequently, patients with TNBCs are currently the subgroup with the worst outcome [8, 9]. Several phase II trials evaluating the role of new biological agents in patients with TNBC have been recently carried out with quite disappointing results. Therefore, there is a worrying lack of phase III registration trials with new molecules in these patients.

The development of high-throughput genomic methods, and the implementation of bioinformatic tools, has revealed the heterogeneity of breast cancer subtypes. These approaches have resulted in profound changes in our understanding of TNBC, and the awareness that this operational term comprises a spectrum of different entities with distinct molecular characteristics that can potentially be targeted in the clinic [10–12].

In this review, we tried to focus on the great diversity that exists between different patients carriers of a so called Triple Negative Tumor. This inability to “*reductio ad unum*” must be given due consideration when we are going to plan the therapeutic strategy in the single woman with TNBC; and above all should be kept in mind when we do clinical research, as the patient with her tumor, rather than the experimental drug, should be the focus of each clinical study.

## THE BIOLOGY OF TNBC

The TNBC is characterized by a quite heterogeneous immunohistochemical phenotype. The majority of TNBCs are high grade, invasive ductal carcinomas, not otherwise specified (ICD-NOS) histology. However, a significant proportion (40%- 100%) of other relatively rare histotypes (medullary and metaplastic carcinomas [13–15], adenoid cystic carcinoma, and apocrine carcinoma [16–18] can also not express ER, PR, and HER2.

TNBC are characterized by a marked degree of nuclear pleomorphism, lack of tubule formation, and high mitotic rates. A substantial proportion of the cases display brisk lymphocytic infiltrate, areas of central necrosis, and pushing borders [19]. Medullary features, as well as areas of focal metaplastic differentiation in the form of squamous and spindle cells, can be found in a subset of these tumors [20–22].

It’s been over a decade since Perou et al published their pioneering article categorizing breast cancer by gene expression profiling into intrinsic subtypes. The analysis led to the discovery and identification of four distinct molecular subtypes of breast cancer with RNA expression profiles [23] (Table 1). These subgroups are characterized by substantial differences in the expression of molecular markers. The luminal A and B subtypes highly express genes normally associated with breast luminal cells, the third subtype is named basal-like because of the expression of genes typically active in breast basal epithelial cells, and the last one, the HER2 subtype, was so defined since overexpressing human epidermal growth factor receptor 2. As opposed to the others, the basal-like subtype is characterized by low expression of ER- and HER2- related genes and clinically is usually, but not always, ER/PR negative and lacking HER2 overexpression (Triple Negative). The basal-like subtype was identified as particularly aggressive, and was associated with the expression of Cytokeratins-5 and 6 (CK5/6), CK14, and CK17; P-cadherin; p53; and epidermal growth factor receptor (EGFR)[24]. Mutations and genomic deletions in tumour protein 53 (TP53) and retinoblastoma 1 (RB1) are common in this subtype, along with a high proliferation index. Although most TNBCs classify as the basal-like subtype, the terms TNBC and basal-like breast cancer are not synonyms; approximately 20% to 30% of clinical TNBCs are not basal-like by microarray analysis, and a not negligible number of basal-like breast cancers express ER/PR or HER2 [12, 20].

**Table 1:** Classification subtypes of breast cancer: classification of breast cancer by gene expression profiling into intrinsic subtype

	**Basal-Like**	
	Gene of breast basal epithelial cell,	
	Low expression of ER and HER2.	
**BREAST CANCER**	**Luminal–Like**	**Luminal A**
	Gene of breast luminal cell.	**Luminal B**
	**HER2**	
	Overexpression of Epidermal Growth Factor Receptor-2.	

Several newer studies have refined our understanding of TNBCs. The Cancer Genome Atlas (TCGA) Research Network analysed primary breast cancers using 6 platforms that included: genomic DNA copy number arrays, DNA methylation, exome sequencing, messenger RNA arrays, microRNA sequencing, and reverse-phase protein arrays [25]. By integrating information across platforms, TCGA analysis demonstrated that the most frequent loss-of-function and gain-of-function alterations in TNBC involve genes associated with DNA damage repair and phosphatidylinositol 3-kinase (PI3K) signalling pathways, respectively. Alterations in DNA damage-repair genes include loss of TP53, RB1, and BRCA1 function [26]. Aberrant activation of the PI3K pathway occurs because of loss of negative regulators, such as the lipid phosphatases (phosphatase and tensin homolog (PTEN) or inositol polyphosphate-4-phosphatase type II, (INPP4B) or activating mutations in PIK3 catalytic subunit A (PIK3CA) along with other genes in the PI3K/target of rapamycin (TOR) signaling network [27].

In another study, Shah and colleagues sequenced and analysed more than 100 TNBC tumours at the time of diagnosis and confirmed the high rate of TP53 mutations, although 12% of tumours did not show somatic mutations in any established driver genes, suggesting that primary TNBCs are mutationally heterogeneous from the outset [10, 28]. Tumours arising in carriers of BRCA1 have many similarities to basal-like sporadic breast tumours, namely a high frequency of TP53 mutations, high tumour grading, and triple negative phenotype. Basal keratins are expressed by both sporadic basal-like tumours and tumours with BRCA1 mutations, and both groups cluster together by gene expression profiling [29]. Other studies support these data, in which familial BRCA1 breast cancers have shared features with a subset of sporadic tumours, indicating a similar aetiology [30]. Hallmarks of this “BRCAness” include basal-like phenotype (associated with the BRCA1 phenotype but not with the BRCA2 phenotype), ER negativity, EGFR expression, TP53 mutations, loss of RAD 51 recombinase (RAD51) focus formation, extreme genomic instability, and sensitivity to DNA-cross-linking agents.

This intrinsic genomic instability in TNBCs and BRCA-associated breast cancers is likely a result of deficient DNA repair and may lead to the success of some chemotherapy regimens [31]. The translational strategy for this group of tumours with “BRCAness” is the design of rational clinical trials that investigate the role of chemotherapy and biologic agents targeting DNA repair defects.

## ANALYZING THE MOLECULAR HETEROGENEITY OF TNBC

The so called TNBC includes, from a genetic point of view, different molecular subtypes. Lehmann et al. [32], by analyzing gene expression profiles from 21 breast cancer datasets, identified six different TNBC sub-types displaying unique gene expression patterns including two basal-like (BL1 and BL2), an immune-modulatory, a mesenchymal (M), a mesenchymal stem-like (MSL), and a luminal androgen receptor (LAR) subtype (Table 2).

**Table 2:** Triple Negative Molecular classification subtypes: By analyzing gene expression profiles of TNBC

		**Basal like Type 1:**
	**Basal Like (BL)**	DNA Damage Response and Cell Proliferation.
	• Proliferation-related genes,	
	• Genes involved in the DNA damage response.	**Basal like Type 2:**
		TP63, EGFR and MET Signaling.
		**Mesenchymal Like**
**Triple Negative**	**Mesenchymal**	EMT, Wnt, TGFβ, IGF1FR, Notch, Cell Proliferation.
ER/PR/HER2 Negative Expression	• Lower proliferation,	
	• Gene expression patterns associated with epithelial-to-mesenchymal transition.	**Mesenchymal Like stem-cell**
		EMT, Wnt, TGFβ, MAPK, Rac, PI3K, PDGF.
		**LAR Type 1:**
	**Luminal Androgen Receptor (LAR)**	AR Signaling, FOXa1 and ERBB4 Signaling,
	• Higher expression of genes involved in androgen receptor signaling.	Luminal A / ER negative.
		**LAR Type 2:**
		AR Signaling,
		Luminal B / ER negative.

It’s includes, from a genetic point of view, different molecular subtypes: Two basal-like (BL1 and BL2), an immunomodulatory (IM), a mesenchymal (M), a mesenchymal stem-like (MSL), and a luminal androgen receptor (LAR) subtype.

Those cancers with BL1 phenotype are enriched not only for proliferation-related genes, but also for the expression of genes involved in the DNA damage response. BL1 cancer cell lines are enriched for sensitivity to specific DNA-damaging agents such as platinum analogs [33, 34]. The BL2 subtype, in contrast, is enriched only for genes related to growth factor signaling. From an immunohistochemical point of view basal-like breast cancers typically express basal cytokeratins such as CK5/6, CK17 as well as cadherin, and epidermal growth factor receptor (EGFR); however, there are no phenotypically patterns which *“per se”* allow us to define basal-like breast carcinoma. TNBCs expressing the basal markers EGFR and CK5/6 have the worst prognosis within the TNBC subtype. Expression of other ‘basal’ markers has also been associated with a poorer outcome, Thike et al [5] evaluated the association of ‘basal’ markers with overall survival (OS) and disease-free survival (DFS) in a large cohort of 653 TNBC, and found a strong correlation between CK17 and CD117 positivity and poor OS.

Mesenchymal-like TNBC subtypes have been further split into two subtypes: mesenchymal-like and mesenchymal stem cell-like. Both subtypes are so named because of enrichment for gene expression patterns associated with epithelial-to-mesenchymal transition [35, 36]. The mesenchymal stem cell-like subtype, in particular, describes a similar group of cancers previously described as claudin-low, that have a lower proliferation, and are enriched for the expression of genes associated with a cancer stem cell-like (or tumor-initiating cell-like) phenotype [37, 38].

The LAR subtype was characterized by higher expression of genes involved in androgen receptor signaling. Androgen receptor mRNA was expressed at an average of 9-fold higher level in this as compared to the other subtypes [39]. Interestingly, LAR subtype belongs to either luminal A or luminal B intrinsic subtype despite being negative for ER expression. The finding of LAR makes it conceptually possible endocrine treatment for at least a proportion of TNBC patients [40].

TNBC and basal-like breast cancer show considerable overlap with BRCA1 mutated tumors [41]. BRCA1 is an important tumor suppressor gene that plays a crucial role in DNA repair, its lack is observed in 5% of all of breast cancer patients [42]. Basal-like breast cancers have been associated with “BRCAness”, which is characterized by high tumor grade, lymphocytic infiltrate, pushing margins, ER and HER2 negativity, TP53 mutations, c-myc amplification, and multiple chromosome abnormalities [43]. BRCA1/2 mutation is very uncommon in sporadic breast cancer [44–46], however, a quite high incidence (about 20%),of germ-line mutations in BRCA1 or 2 has been reported in patients with TNBC [46].

## CURRENT SYSTEMIC TREATMENT OF TRIPLE NEGATIVE BREAST CANCER

### Early-stage disease

The only opportunity to treat early-stage TNBC with curative intent is systemic chemotherapy, because there are currently no approved targeted treatments, like endocrine or HER2-directed therapy, to ameliorate baseline risk. As such and in compliance with guidelines put forth by both the National Cancer Comprehensive Network and the St. Gallen International Expert Consensus, it is common and appropriate for oncologists to prescribe an anthracycline/taxane-based adjuvant chemotherapy in patients with early TNBC.

Randomized trials provided evidence for the incremental benefit of dose-dense administration of chemotherapy in patients with TNBC [47]. Such incremental progress now provides a greater than 50% reduction in the odds of recurrence and a similar reduction in odds of death for patients with TNBC. The role of anthracyclines in patients with early TNBC has recently been questioned. A retrospective analysis from the MA5 Trial, randomly assigning patients to receive either CMF or CEF adjuvant chemotherapy, showed an increased 5-year DFS for the former (71% *versus* 51%, respectively) among patients with Basal-like breast cancer (BLBC); the test for interaction between Basal-like phenotype and treatment arm reached borderline significance (*P* = 0.06) indicating that patients with TNBC may not derive a particular benefit from anthracyclines [48]. Although these retrospective results challenge the role of anthracyclines in adjuvant therapy for TNBC/BLBC, additional data will be needed for final clarification of this issue.

However, it is also true that “*biology does not trump anatomy*”. A small node-negative TNBC carries a low (15% or less) 5-year risk of recurrence and a proportionally lower benefit of treatment. Using tools, such as *Adjuvant! OnLine*, the mortality risk at 10 years for T1a/bN0 tumours is less than 10%. An observational study of more than 1000 T1a/bN0 TNBCs found excellent prognosis, with 95% remaining free of distant metastasis at 5 years and without a notable difference between those who did and those who did not receive chemotherapy. Taking this into account, it is reasonable to offer adjuvant chemotherapy in node-negative TNBC with a >1 cm tumour size; a balanced discussion could be needed for 0.6 cm to 1.0 cm tumours; and no adjuvant chemotherapy should be administered in breast tumours 0.5 cm or less (T1a). As with other subtypes of breast cancer, adjuvant anthracycline/taxane-based chemotherapy is recommended in patients with lymph node positive disease (N1 or greater), regardless of primary tumour size.

A great change has taken place in recent years in the management of early breast cancer. It consists in delaying the surgery in order to assess *“in vivo”* the efficacy of the medical treatment chosen for that patient. This approach, called primary/neoadjuvant therapy, was initially adopted when there was a need to reduce the tumour mass and avoid radical surgery. Nowadays, the neoadjuvant treatment has become much more popular and is widely adopted both in clinical practice and in the research field. The neoadjuvant setting represents a formidable tool for the evaluation of new molecules, because it allows a better understanding of the interactions between the drug, the tumour and the host.

**Figure 1:**
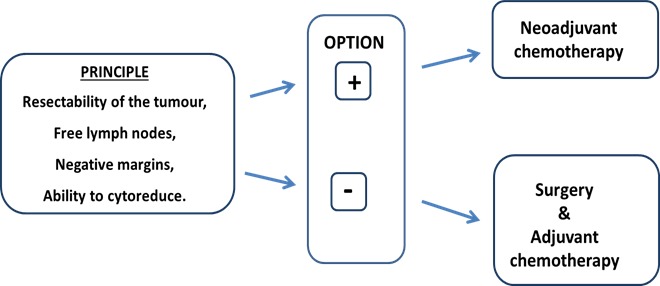
Principle of neoadjuvant chemotherapy strategy

The principles that govern the decision to proceed with neoadjuvant *versus* adjuvant chemotherapy are similar between TNBC and other subtypes of breast cancer. These principles are largely driven by (1) resectability of the primary tumour and lymph nodes to achieve negative margins and (2) the ability to cytoreduce a breast cancer to facilitate breast conservation, as opposed to a mastectomy. However, there is a *“condicio sine qua non”* to justify the choice of a neoadjuvant chemotherapy, namely that the patient has such a risk of relapse with the surgery alone to make it not possible to avoid an adjuvant chemotherapy (Figure 1).

Basal-like/TNBC has consistently been shown more sensitive to neoadjuvant chemotherapy (i.e., higher pCR rates) than luminal breast cancers. The rate of pathologic complete responses, defined as the absence of invasive tumour in both breast and axilla, may exceed 30% with the best chemotherapy regimens, compared to percentage of around 10% achievable in the luminal tumors as a whole (being the pCR almost anecdotal in luminal A).

Collectively, however, TNBC patients experience poorer overall outcomes compared with other breast cancer subtypes. The poorer prognosis of basal-like/TNBC has been explained by a higher likelihood of relapse in those patients in whom pCR was not achieved and has been termed “*the triple-negative paradox”*. Using pCR rates for patients with TNBC as an endpoint, investigators are evaluating additional chemotherapies and targeted agents in the neoadjuvant setting. Platinum agents and bevacizumab are the most interesting drugs currently under investigation in TNBC patients undergoing neoadjuvant/adjuvant therapy [49].

Platinum analogs attack cancer cells by inducing double-stranded DNA breaks. As single agents, they have limited efficacy in heavily pre-treated metastatic breast cancer, but greater activity has been seen in BRCA-mutation carriers, with pCR rates of 70% in small neoadjuvant trials. BRCA-mutated and sporadic TNBC have similar biologic characteristics and mRNA gene expression patterns, motivating further study of platinums in this subtype. Although single-agent cisplatin yielded few pCRs in sporadic TNBC, pilot studies of the addition of cisplatin or carboplatin to standard neoadjuvant chemotherapy have reported more promising results [50].

Bevacizumab binds and inactivates Vascular Endothelial Growth Factor 1, believed to support the growth and maintenance of tumour neovasculature necessary for survival and metastasis. In 2012 the results of two very large randomized studies addressing the role of bevacizumab in the neoadjuvant treatment of HER2 negative breast cancer were published in the New England Journal of Medicine [51, 52]. The German Study [52] showed a clear advantage with bevacizumab addition only in patients with TNBC; conversely the American Trial [51] showed a greater effect of bevacizumab treatment in ER-positive patients. The favorable prognostic role of bevacizumab has been strongly questioned by the results of the BEATRICE Trial [53]; in this large phase III randomized study the addition of bevacizumab to standard adjuvant chemotherapy failed to improve Disease-free survival in patients with early TNBC.

The CALGB (Cancer and Leukaemia Group B) 40603 Trial was designed to examine the impact of adding carboplatin and/or bevacizumab to conventional neoadjuvant chemotherapy in TNBC. Four hundred and forty-three patients with stage II to III TNBC received paclitaxel 80 mg/m^2^ once per week for 12 weeks, followed by doxorubicin plus cyclophosphamide once every 2 weeks for four cycles, and were randomly assigned to concurrent carboplatin (area under curve 6) once every 3 weeks for four cycles and/or bevacizumab 10 mg/kg once every 2 weeks for nine cycles. The addition of either carboplatin (60% *vs.* 44%; *P* = 0.0018) or bevacizumab (59% *vs.* 48%; *P* = 0.0089) significantly increased pCR in the breast, whereas only carboplatin (54% *vs.* 41%; *P* = 0.0029) significantly raised pCR in both breast and axilla [54].

In the GeparSixto Trial 315 stage II-III TNBC patients were randomized to receive neoadjuvant anthracycline-taxane-bevacizumab therapy with or without carboplatin. The pCR rate was significantly higher in the carboplatin arm (53.2% *vs*. 36.9%; *p* = 0,005) [55]. The same authors evaluated the correlation between Tumour Infiltrating Lymphocytes (TILs) and response to chemotherapy. Increased levels of stromal TILs predicted a higher pCR rate in the whole population. However, the advantage of carboplatin arm was much higher in patients with higher stromal TILs (74% *vs* 43%; *p* = 0.0005) than in the others (46% *vs*. 34%; *p* = 0.08) [56].

### Advanced disease

In spite of great excitement in the recent past with potential novel drugs, like PARP inhibitors and bevacizumab, no targeted therapy in the metastatic setting is available at this time. Conventional treatment of metastatic TNBC begins with cytotoxic chemotherapy, of which there are approximately 14 single agents and approximately 8 doublets listed in the treatment of HER2-negative, recurrent, or metastatic breast cancer according to the National Cancer Comprehensive Network (www.NCCN.org). Choice of palliative cytotoxic regimen is no different in TNBC than in other subtypes, with options of poly-chemotherapy generally reserved for symptomatic or rapid visceral progression and sequential single agents for asymptomatic, stage IV disease.

A recent randomized phase III study, CALGB 40502, confirms that weekly paclitaxel is the optimal first-line regimen in the TNBC subset, when compared with the more modern microtubule-directed agents, nab-paclitaxel or Ixabepilone [57].

The use of bevacizumab in combination with chemotherapy has been shown to improve both response rates and Progression Free Survival butthis does not translate into a meaningful prolongation of median survival [58].

## THE FUTURE OF THE TREATMENT OF TNBC

Ongoing preclinical and clinical efforts are focused on the development of more refined strategies to control advanced TNBC beyond that of cytotoxic chemotherapy. In the recently reported TCGA analysis, the most commonly mutated genes and pathways in basal-like/TNBC were the tumor suppressor gene TP53 (approximately 80% mutated) and loss of RB1 (tumor suppressor gene) and BRCA1 (DNA repair gene) function as well as PIK3CA (approximately 9% compared with approximately 30%-49% in luminal A and B breast tumors, respectively). Other plausible drug targets identified through this comprehensive analysis included FGFR1, FGFR2, IGFR1, KIT, MET, PDGFRA as well as angiogenesis and/or drugs that become activated under hypoxic conditions. Here in we try to briefly outline some potential therapeutic strategies.

### “Scraping the bottom of the barrel” with chemotherapy

As previously mentioned, the progress of chemotherapy in the last decades have produced an impressive prognostic gain in TNBC patients. Some issues remain open, and first of all if it is still possible to make further improvements “*scraping the bottom of the barrel*” of chemotherapy. The cytotoxic drugs around which revolves the therapeutic strategy are the alkylating agents, anthracyclines and taxanes. Capecitabine is an oral drug of relatively recent introduction, which is commonly used in advanced disease. Some authors have suggested that its use in early disease in addition to the three categories mentioned above could lead to a substantial improvement in the prognosis. The FinXX Study [59] evaluated the addition of capecitabine to a standard anthracycline-taxane based adjuvant therapy in patients with high risk breast cancer. Although the addition of capecitabine failed to achieve a significant advantage in the whole population, it significantly improved breast cancer-specific survival (HR, 0.64; 95% CI, 0.44 to 0.95; *P* = 0.027) and RFS in women with triple-negative disease. Another burning issue is the use of dose-dense or dose-intensified chemotherapy with or without the addition of platinum compounds. The two neoadjuvant studies we mentioned above, showed that the addition of carboplatin to an anthracycline-taxane dose-dense chemotherapy results in a substantial increase in pCRs [54, 55].

The ETC (epirubicin 150mg/m² every 2 weeks for 3 cycles followed by paclitaxel 225 mg/m² every 2 weeks for 3 cycles followed cyclophosphamide 2000 mg/m² every 2 weeks for 3 cycles) regimen represents the paradigm of sequential non platinum-including dose-intensified chemotherapy and proved to be a highly effective adjuvant therapy for TNBC patients.

The ongoing GeparOcto Trial aims to evaluate the role of platinum *“per se”* by comparing ETC with the weekly carboplatin/paclitaxel/non-pegylated liposomal doxorubicin regimen as neoadjuvant treatment of high-risk TNBC patients [60].

The long-standing controversy about the role of platinum compounds (if they can be considered equivalent) in the treatment of TNBC is far from over. A recent study seems to help clarify some of the terms of the issue. The TBCRC009 was a Multicenter Phase II Clinical Trial of Platinum Monotherapy in Metastatic Triple-Negative Breast Cancer, which aimed at highlighting the relationships between biomarkers and therapeutic efficacy. Patients (*N* = 86; 69 as first-line therapy) received cisplatin or carboplatin. ORR was 25.6% and was numerically higher with cisplatin (32.6%) than with carboplatin (18.7%). ORR was 54.5% in the 11 patients with germline BRCA1/2 mutations. In the 66 patients without BRCA1/2 mutations, exploratory analyses showed that a BRCA-like genomic instability signature discriminated responding and non-responding tumours (mean homologous recombination deficiency-loss of heterozygosity/homologous recombination deficiency-large-scale state transitions (HRD-LOH/HRD-LST) scores were 12.68 and 5.11, respectively). According to these data, platinum agents should be recommended in TNBC patients, especially in those with germline BRCA1/2 mutations or with high HRD-LOH/HRD-LST score [61].

### Targeting angiogenesis

Anti-angiogenic strategies seemed promising based on preclinical data in TNBC models; however, a pooled analysis, of 3 randomized first-line metastatic studies of bevacizumab added to chemotherapy (E2100, AVADO, and RIBBON-1) demonstrated improvement in PFS but no impact on OS in HER2-negative patients overall or in the TNBC subset [58]. The studies conducted in the neoadjuvant setting could not rule out a potential positive effect of bevacizumab, however, the results of the large adjuvant BEATRICE Study does not seem to justify the addition of bevacizumab to chemotherapy in early TNBC patients. Biomarkers predictive of response to bevacizumab are urgently needed, although they are probably difficult to identify.

Ramucirumab is a human immunoglobulin G1 antibody that binds vascular endothelial growth factor receptor-2 and blocks ligand-stimulated activation. The ROSE/TRIO-012 Trial evaluated ramucirumab with docetaxel in HER2 negative locally-advanced, or metastatic breast cancer. Both median PFS (9.5 *vs*. 8.2 months; *p* = 0.077) and OS (27.3 *vs*. 27.2 months; *p* = 0.915) were not significantly better in patients treated with ramucirumab plus docetaxel as compared to patients who received placebo plus docetaxel [62].

### Inhibitors of poly-ADP-ribose polymerase

In those patients with germline BRCA1 or BRCA2 mutations, PARP inhibition remains a promising avenue; however, this approach remains available only in clinical trials, and reports from small studies have failed to demonstrate a similar outcome in sporadic TNBC. The PARP inhibitor, Olaparib, revealed unconfirmed responses only in BRCA1/2 carriers, with no responses among patients with sporadic TNBC [63]. A phase II study evaluating the combination of temozolomide with another PARP 1/2 inhibitor, Veliparib (ABT-888), enrolled 41 patients with metastatic breast cancer (approximately 50% TNBC); response rate across the entire population was 7%; however, an exploratory analysis revealed that responses were essentially limited to BRCA1/2 carriers. PFS was 5.5 months for BRCA mutation carriers and 1.8 months in non-carriers, suggesting that the benefit from PARP inhibition was largely derived from those harboring mutations in DNA repair, namely through the BRCA pathway [64]. Identifying non-BRCA associated TNBC tumors with similar phenotype and DNA damage repair defect with potential to benefit from PARP inhibition, with or without chemotherapy, remains a subject of intense and ongoing research.

The results of a large phase III study, however, have very dampened the enthusiasm around the use of the PARP-inhibitors in metastatic TNBC. Five hundred nineteen patients with stage IV TNBC who had received no more than two previous chemotherapy regimens were randomly allocated to gemcitabine/carboplatin (GC) alone or GC plus Iniparib. The co-primary end points were OS and PFS. In the primary analysis, no statistically significant differences were observed for both end points. An exploratory analysis showed that patients in the second-/third-line had improved OS (HR = 0.65; 95% CI, 0.46 to 0.91) and PFS (HR = 0.68; 95% CI, 0.50 to 0.92) with GCI [65].

The hypothesis that an earlier use of Iniparib could result in a better outcome, is the basis of its use in the neoadjuvant setting. The carboplatin-gemcitabine-iniparib combination was recently tested as neoadjuvant therapy for Triple-Negative and BRCA1/2 Mutation-Associated Breast Cancer. All patients underwent comprehensive BRCA1/2 genotyping, and homologous recombination deficiency was assessed by loss of heterozygosity (HRD-LOH) in pre-treatment core breast biopsies. Among 80 patients, 19 patients (24%) had germline BRCA1 or BRCA2 mutations. Overall pathologic complete response rate in the intent to treat population was 36%. Mean HRD-LOH scores were higher in responders compared with non-responders (*P* = 0.02) and remained significant when BRCA1/2 germline mutations carriers were excluded (*p* = 0.021). If these results will be confirmed in further larger trials, the administration of Iniparib could be recommended for patients with either BRCA1/2 mutated or sporadic triple-negative breast cancer lacking a BRCA1/2 mutation, but with an elevated HRD-LOH score [34].

A large 3-arm randomized trial (NCT02032277) is ongoing which aims at evaluating the addition of either carboplatin or carboplatin and ABT-888 to a standard paclitaxel-AC regimen as neoadjuvant therapy of early TNBC patients. The design of the present study is very ambitious, however, the choice to include unselected TNBC patients and the planned sample size (about 200 patients per arm) could make very problematic the detection of statistically significant differences between the 3 arms, especially with regard to Disease-free and overall survival [66].

### Inhibition of epithelial growth factor receptor

Although TNBC lacks ER and HER2 expression, expression of the epithelial growth factor receptor (EGFR), HER1, has been demonstrated in TNBCs at both the gene and protein level. Several studies have evaluated the benefit of adding the EGFR-targeted monoclonal antibody, cetuximab, to platinum-based chemotherapy to treat advanced TNBC with modest results.

The TBCRC-001 Study evaluated treatment with cetuximab as a single agent or combined with carboplatin among 102 patients with advanced TNBC. Response rates for cetuximab as a single agent, combined with carboplatin at progression after monotherapy, or in combination from the onset of treatment, were 6%, 16%, and 17%, respectively [67].

The BALI-1 Study reported a doubling of response rates by combining cetuximab with cisplatin compared with cisplatin alone in TNBC (20% *vs* 10.3%); however, the duration of response was short in both arms, the median PFS being only 3.7 and 1.5 months, respectively [68, 69]. Although there was initial enthusiasm and strong preclinical rationale for the incorporation of EGFR-based therapy into systemic therapy for advanced TNBC, translation of this approach clinically has resulted in only modest improvements in outcome, possibly due to heterogeneity of disease and compensatory alternate signaling in the cancer cells.

Biomarkers predictive of response to this targeted therapy or combinatorial strategies may be needed to improve the efficacy of this approach.

### Inhibition of AR signaling

The luminal AR subtype of TNBC is sensitive to androgen deprivation in preclinical studies, making AR signaling in ER-negative breast cancer an intriguing potential target. In the recent TBCRC 011 Phase II Trial, more than 450 hormone receptor-negative (primarily TNBC) patients were screened, of which approximately 10% had AR expression. Single-agent Bicalutamide in these patients yielded a clinical benefit in 19%. Continued study of AR pathway inhibition in advanced TNBC albeit the small subset that may be driven by the AR pathway is warranted as the era of personalized medicine is approached [70].

It has been recently reported that other non-LAR molecular subtypes of TNBC are critically dependent on AR protein. Indeed, AR inhibition significantly reduces baseline proliferation, anchorage-independent growth, migration, and invasion and increases apoptosis in four TNBC lines (SUM159PT, HCC1806, BT549, and MDA-MB-231), representing three non-LAR TNBC molecular subtypes (mesenchymal-like, mesenchymal stem-like, and basal-like 2). *In vivo*, enzalutamide was able to significantly decrease viability of SUM159PT and HCC1806 xenografts [71]. A Phase II Study (NCT01889238) is now ongoing which aims at evaluating the safety and efficacy of Enzalutamide in patients with advanced, androgen receptor-positive, and TNBC [72].

### Inhibition of the PI3K, MEK, CHK, and HDAC pathways

As identified in TCGA, activation of the PI3K, either directly via PI3KCA mutations or indirectly via PTEN and/or INPP4B loss, has been identified as important in TNBC/basal-like breast cancer. In Preclinical Study, inhibition of the PI3K pathway results in TNBC cell growth arrest; several small molecule inhibitors of the PI3K (and downstream mTOR pathway) are in development. Several phase I/II studies (NCT01623349, NCT01629615, NCT01918306) are ongoing which aim at evaluating the role of the PI3K inhibitors (either alone or combined with platinum or PARP inhibitors) in TNBC patients.

An open label Phase II randomized study [73] has recently been carried out in women with TNBC, which evaluated the addition of the mTOR inhibitor everolimus to a standard neoadjuvant chemotherapy. There was downregulation of the mTOR pathway at 48 h in the everolimus arm; however, that did not result into a significant increase of pCR rate (30.4% *vs*. 25.9%; *p* = 0.76).

TCGA analysis has also identified high rates of p53 (tumor suppressor gene) mutations in TNBC/basal-like breast cancer. In the absence of p53 function, cells in need of DNA damage repair rely on checkpoint kinase 1 (Chk1) to arrest the cell cycle and push potentially defective cells toward apoptosis; p53-deficient mouse models of breast cancer are sensitive to Chk-1 inhibition. Chk-1 inhibitors have, therefore, become an attractive potential target for the treatment of TNBC harboring p53 mutations [74]. Unfortunately, the preliminary clinical data have dampened the enthusiasm derived from the preclinical studies. UCN-01, a non-selective Chk1 inhibitor, has recently been tested in combination with Irinotecan in heavily pre-treated metastatic TNBC patients. The overall response rate was only 4%, with a clinical benefit rate of 12% [75].

The inhibition of mitogen-activated protein/extracellular signal regulated kinase (MEK), in combination with PI3K/mTOR inhibition, has shown activity in a TNBC/claudin-low genetically engineered mouse model [76]; a window study of MEK inhibition (GSK1120212) is ongoing to evaluate dynamic reprogramming of the kinome in patients with TNBC to further identify pathways of resistance. Finally, epigenetic regulation of gene expression has been a hot topic in TNBC for several years. An inhibitor of the Histone Deacetylase (HDAC) pathway (Panobinostat) has been shown to decrease cell growth in TNBC cell lines as well as tumorigenesis *in vivo* and may soon make its way into the clinic [77, 78].

The randomized Phase II Study TBCRC-008, which evaluated, in an unselected TNBC group, the addition of Vorinostat to preoperative carboplatin and nanoparticle albumin-bound paclitaxel did not show a significant increase of pCR rate in the experimental arm (27.6% *vs*. 26.7%) [79].

Several publications have suggested that Histone Deacetylase Inhibitors (HDACs) could reverse the repression of oestrogen receptor alpha (ERα) in triple-negative breast cancer (TNBC) cell lines, leading to the induction of a functional protein. The results of a recent study, seem to question the intriguing hypothesis that HDAC could reverse the repression of oestrogen receptor alpha in TNBC [70].

## CHALLENGING THE PARADIGMS OF CLINICAL TRIALS IN TNBC

Over 30 years of clinical trials testing adjuvant chemotherapy, have radically changed the fate of patients with TNBC. However, this model of clinical research raises increasing criticism since it requires a disproportionate expenditure of time and patients before allowing registration of new drugs. In the last decade there has been an exponential growth of neoadjuvant clinical trials, since the correlation between pCR (or minimal residual tumor burden) and long-term survival, could allow a more rapid evaluation of new therapies without the need of long-term follow-up to demonstrate survival benefits. FDA recently ruled that it may grant accelerated approval of a new drug provided that it had shown to significantly improve the rate of pathological complete responses.

The need to speed up the process of clinical evaluation of new drugs has led all players in the fight against cancer to design a new comprehensive platform for the evaluation of new anticancer molecules. Investigation of serial studies to predict your therapeutic response with imaging and molecular analysis (I-SPY 2) is a collaborative effort among academic investigators, the National Cancer Institute, the US Food and Drug Administration, and the pharmaceutical and biotechnology industries under the auspices of the Foundation for the National Institutes of Health Biomarkers Consortium.

**Figure 2:**
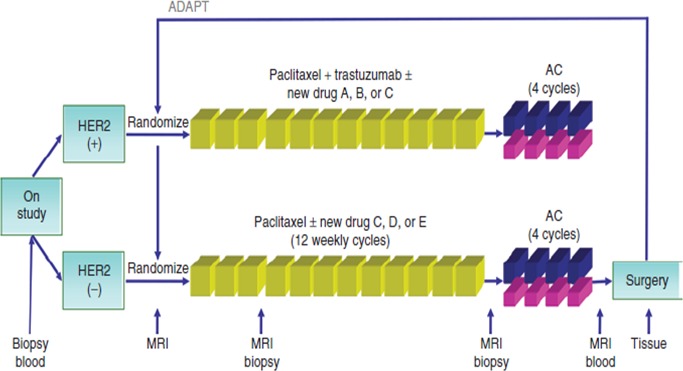
I-Spy trial

I-SPY 2 was developed to allow the activity of drugs to be assessed much earlier in the research process, potentially enabling drugs to be developed and approved using fewer patients, less time and far fewer resources. The goal is to shave several years and hundreds of millions of dollars off the current process. The I-SPY 2 TRIAL focus on treatment in the neoadjuvant setting [80] (Figure 2). All patients receive the current standard of care and most participants receive one investigational drug. This model seems to respond perfectly to the needs of the scientific community, but it could not be the most appropriate response to the expectations of patients. In I-SPY 2 as well as in most of the newest clinical investigations the patient is treated as a *“living laboratory”* where to test new drugs as quick as possible. Unfortunately, this model, although in a more sophisticated way than in the past, is still focused on the fate of the drug rather than on the needs of the patient. What is of interest to the patient is to receive a treatment that is both highly effective and little toxic. This does not necessarily mean to receive the newest and/or most aggressive available therapy.

Let’s imagine an happy ending for the evaluation of the experimental drug x. The preliminary phase II and the subsequent phase III confirmatory trial show that the combination of x with an aggressive chemotherapy results in a 20% increment of the expected pCR rate (30% to 50%). This very favorable scenario implies that we have over-treated those 30% who were expected to achieve a pCR with aggressive chemotherapy alone. Moreover, the choice of an aggressive chemotherapy, as a standard treatment, might have caused an unnecessary toxicity in those patients expected to achieve a pCR even with a more gentle chemotherapy. Finally, this model does not offer additional therapeutic options to the 50% of patients who have failed to obtain the pCR.

**Figure 3:**
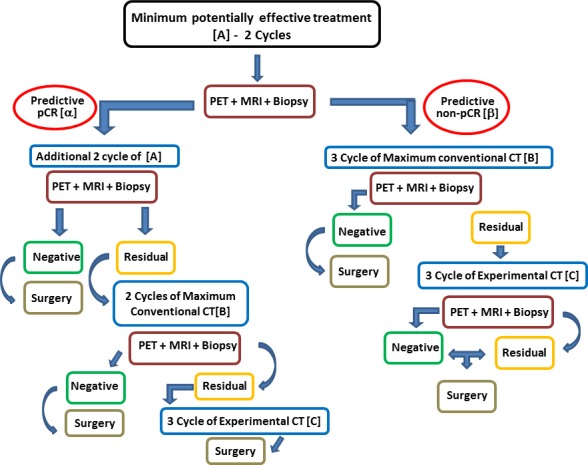
proposed model **α.** – Predefined drop of SUV Max and K-Trans + proliferation (Ki67) + predefined increment of apoptosis (caspase). **β.** – Failure in achieving at least one of the above of the above α criteria; **A.** – Caelyx + Xel/ Vin or CMF or AR Inhibitor. **B.** – Maximum Conventional Treatment (platinum-anthracycline-taxane for carriers of BRCA1/2 mutation/BRCA-like genomic instability signature, dose-dense anthracycline-taxane for the other). **C.** – Different investigational approach according to molecular definition (Example: 1) Carbo-GEM + Parp-Inhibitor for BRACness subtype or Best Standard CT+ AR Inhibitor for LAR subtype)).

We propose a model that does not provide just one option to the patient (i.e. aggressive chemotherapy + experimental drug), but rather offers a range of options ranging from the minimum therapy that is potentially effective up to the maximum theoretically possible treatment (including the use of one or more investigational drugs) (Figure 3). The preliminary analysis of the biology of the tumor allows to define at least three main therapeutic options: the least potentially effective treatment (CMF for basal-like, androgen inhibitor for LAR; etc…), the most effective conventional treatment (platinum-anthracycline-taxane for carriers of BRCA1/2 mutation/BRCA-like genomic instability signature, dose-dense anthracycline-taxane for the others), the combination of the second option with at least one experimental drug potentially active in that particular tumor (PARP/VEGF/PI3K/HDAC/etc-antagonist) (Table 3). The route includes three stages before the surgery. After the molecular definition of the tumor (BL1- 2 or IM etc...) and the baseline assessment of both hystologic (proliferation, apoptosis, hypoxia etc.) and imaging (FdG-PET, and dynamic enhanced MRI) parameters, the route starts with the administration to the patient of the least potentially effective treatment based on the data available. A first check of the effectiveness of the treatment chosen will be made after about one month (1 or 2 cycles if chemotherapy is the chosen starting treatment) and will be based on the reassessment of those features which are considered valid surrogates of the pCR. If the test predicts a pCR the patient continues the same therapy until the second check-point, otherwise she switches to the predefined maximum conventional treatment. In case of prediction of pCR at the second check, the patients continues the ongoing treatment until the surgery. If a non-pCR is predicted at the second check, treatment will be switched to maximum conventional chemotherapy alone or combined with an experimental drug depending on what had happened at the first check-point. A last chance can be given to the patient showing residual disease at surgery, by giving her an additional experimental drug as adjuvant treatment.

**Table 3:** Therapeutic option

	Histological Phenotype	Chemotherapy
**Effective Treatment**	Basal Like	C.M.F.
	Luminal Androgen Receptor	Androgen Inhibitor
	Mesenchimal Like	
**Conventional Treatment**	Basal like	
	Luminal Androgen Receptor	Platinum - Anthracicline - Taxane
	Mesenchimal Like	
**Experimental Drugs**	Basal like	
	Luminal Androgen Receptor	PARP/VEGF/PI3K/HDAC/ etc inhibitor
	Mesenchimal Like	

## CONCLUSIONS

This work provides one of the first substantiations that TNBC is a unique entity characterized not only by adverse prognostic features, but also by a diverse underlying biology against which novel therapeutics should be targeted.

Even today it is still believed that for TNBC are worth the same principles which have proved valid for leukemias and lymphomas (more chemotherapeutic agents/higher doses/shorter intervals). As a matter of fact the long journey, from the rudimentary chemotherapy regimens of the 80s to the current sophisticated chemotherapy approaches, has reduced the mortality by 50%. However, we all understand that chemotherapy has reached its *“Pillars of Hercules”*, therefore we urgently need to identify new targets (where at a first glance does not seem to be anyone), if we want finally overcome that obstacle, and achieve the cure for those patients otherwise expected to relapse when treated with chemotherapy alone. The main lesson to be learned from the most recent findings in molecular biology is that “*one size does not fit all TNBCs*”, and tailored therapy is the “*magic formula*” also for the women bearing a TNBC. We must dispel the myth that all patients with TNBC should receive an aggressive chemotherapy; some types of TNBC could not require any further treatment after surgery, since *“per se”* they have a good prognosis. Other subtypes may be so chemo-sensitive to make even a gentle chemotherapy enough to achieve the cure. Some TNBC patients should not receive chemotherapy since they could be cured with a not standard endocrine treatment (e.g. antiandrogens), or with a standard endocrine therapy (SERMs or Aromatase inhibitors) when combined with drugs which can restore ER expression.

A real Copernican revolution will happen when all of us will become aware that the first is the study of the tumor of that particular patient and then follows the choice of the therapeutic strategy. Even more important in our view is to become aware of the fact that the preliminary definition of the characteristics of the tumor does not exhaust our task. The therapy for each patient can’t be predefined once and for all, but must rather be a therapeutic strategy of *“variable geometry”*, namely it must be so flexible to target the maximum results with the minimum cost. Cancer research of the third millennium cannot continue to be drug- or investigator-centered, rather must put at the center the individual patient. The alpha and omega of our effort should focus on the human being, and each question deriving from the study of the patient with her tumor has to find the proper answer there in the same patient.
